# Supplementary material for: The efficacy of interventions in reducing belief in conspiracy theories: A systematic review
Source: PLoS One. 2023 Apr 5;18(4):e0280902. doi: 10.1371/journal.pone.0280902 (PMC10075392; doi:10.1371/journal.pone.0280902)
Supplement: S1 Table — (DOCX) [file pone.0280902.s001.docx]

| Title | Intervention | Age | Country | Sample (EXP/CTRL) | Female proportion | Data collection |
| --- | --- | --- | --- | --- | --- | --- |
| [46] | Prevention Regulatory Focus | 35.59 ± 10.69 | US | NR | 46.80% | In-person |
| [46] | Promotion Regulatory Focus | 35.59 ± 10.69 | US | NR | 46.80% | In-person |
| [46] | Increasing Perceived Control (Promotion) | 22.93 ± 5.41 | US | NR | 51.10% | In-person |
| [46] | Increasing Perceived Control (Prevention) | 22.93 ± 5.41 | US | NR | 51.10% | In-person |
| [47] | Rationality Priming | 43.53 ± 9.34 | France | 377/385 | 69.20% | Online |
| [41] | Ridiculing Beliefs | 46.43 ± 14.74 | Hungary | NR | 51.10% | Online |
| [41] | Rational counterarguments | 46.43 ± 14.74 | Hungary | NR | 51.10% | Online |
| [41] | Empathetic counterarguments | 46.43 ± 14.74 | Hungary | NR | 51.10% | Online |
| [21] | Fact-based Inoculation | 20.37 ± 1.85 | US | 61/68 | 54% | In-person |
| [21] | Logic-based Inoculation | 20.37 ± 1.85 | US | 57/68 | 54% | In-person |
| [21] | Fact- based Metainoculation | 20.37 ± 1.85 | US | 65/68 | 54% | In-person |
| [21] | Logic-based Metainoculation | 20.37 ± 1.85 | US | 60/68 | 54% | In-person |
| [25] | Analytical priming | 19.54 ± 3.06 | United Kingdom | 58/54 | 58.90% | In-person |
| [25] | Analytical priming | 19.72 ± 4.39 | United Kingdom | 94/95 | 61.90% | In-person |
| [25] | Analytical priming | 33.87 ± 15.05 | United Kingdom | 74/66 | 47.10% | In-person |
| [15] | Pro-conspiracy arguments | 31.73 ± 9.93 | United Kingdom | 55/52 | 36.50% | Online |
| [15] | Anti-conspiracy arguments | 31.73 ± 9.93 | United Kingdom | 52/52 | 36.50% | Online |
| [15] | Anti-conspiracy/conspiracy arguments | 31.73 ± 9.93 | United Kingdom | 50/52 | 36.50% | Online |
| [15] | Conspiracy/anti-conspiracy arguments | 31.73 ± 9.93 | United Kingdom | 51/52 | 36.50% | Online |
| [15] | Anti-conspiracy/conspiracy arguments | 34.02 ± 11.10 | United Kingdom | 64/58 | 54.20% | Online |

**Table 2. Demographic and publication details of studies included in the review**

| **Table 2. continued** | | | | | | |
| --- | --- | --- | --- | --- | --- | --- |
| Title | Intervention | Age | Country | Sample (EXP/CTRL) | Female proportion | Data collection |
| [15] | Conspiracy/anti-conspiracy arguments | 34.02 ± 11.10 | United Kingdom | 53/58 | 54.20% | Online |
| [48] | Conspiracy labelling | 35.38 ± 10.94 | United Kingdom | 67/83 | 40.60% | Online |
| [48] | Conspiracy labelling | 32.28 ± 11.33 | United Kingdom | 404/398 | 40% | Online |
| [27] | Priming Resistance to Persuasion | 18.79 ± 1.22 | France | 41/40 | 97.50% | In-person |
| [27] | Priming Resistance to Persuasion | 21.51 ± 4.57 | France | 94/111 | 84.90% | Online |
| [27] | Priming Resistance to Persuasion | 20.27 ± 1.83 | France | 128/137 | 77.70% | Online |
| [16] | Debunking | 33.39 ± NR | Macedonia | 56/57 | 70.60% | Online |
| [16] | Debunking, motives, fallacy | 33.39 ± NR | Macedonia | 47/57 | 70.60% | Online |
| [40] | High control priming | 36.20 ± 12.24 | New Zealand | 85/108 | 79% | Online |
| [40] | Low control priming | 36.20 ± 12.24 | New Zealand | 79/108 | 79% | Online |
| [40] | High control priming | 37.28 ± 11.66 | New Zealand | 83/116 | 53% | Online |
| [40] | Low control priming | 37.28 ± 11.66 | New Zealand | 72/116 | 53% | Online |
| [40] | Low control priming | 39.14 ± 12.62 | New Zealand | 86/109 | 61% | Online |
| [40] | Low control priming | 40.85 ± 13.44 | New Zealand | 83/109 | 63% | Online |
| [26] | Ostracism priming | 34.27 ± 12.26 | Hong Kong | NR | 71.31% | Online |

| **Table 2. continued** | | | | | | | | | | | |  |
| --- | --- | --- | --- | --- | --- | --- | --- | --- | --- | --- | --- | --- |
| Title | Intervention | | Age | | Country | | Sample (EXP/CTRL) | | | Female proportion | Data collection |  |
| [26] | | Ostracism priming | | 36.05 ± 12.17 | | Hong Kong | | NR | 65.90% | | Online | |
| [26] | | Ostracism priming/self-affirmation | | 20.78 ± 1.70 | | Hong Kong | | NR | 82% | | Online | |
| [26] | | Ostracism priming/no affirmation | | 20.78 ± 1.70 | | Hong Kong | | NR | 82% | | Online | |
| [49] | | Narrative persuasion | | 25.90 ± 6.7 | | Belgium | | 37/41 | 44.40% | | Online | |
| [50] | | Pseudoscience class | | NR | | US | | 208/413 | 47.30% | | In-person | |
| [50] | | Research Methods class | | NR | | US | | 238/413 | 47.30% | | In-person | |

*Note.* NR = Not reported; Sample (EXP/CTRL) = the number of participants in the experimental condition vs. control condition; Data collection = whether data was collected in-person or online.
