# Supplementary figures and images for: The efficacy of interventions in reducing belief in conspiracy theories: A systematic review
Source: PLoS One. 2023 Apr 5;18(4):e0280902. doi: 10.1371/journal.pone.0280902 (PMC10075392; doi:10.1371/journal.pone.0280902)

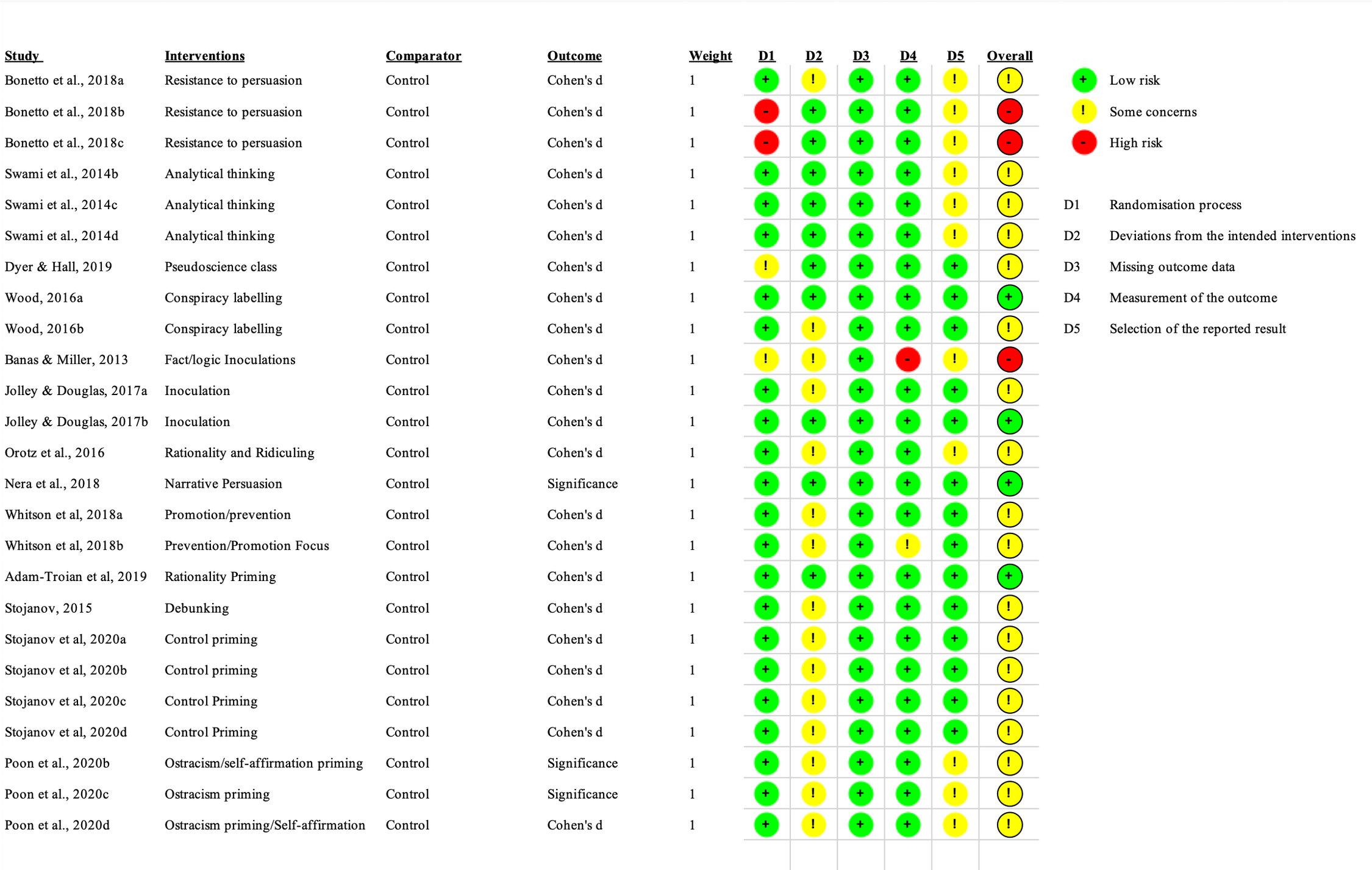

Supplement: S1 Fig — (TIF) [file pone.0280902.s002.tif]
